# Predicting the COVID‐19 mortality among Iranian patients using tree‐based models: A cross‐sectional study

**DOI:** 10.1002/hsr2.1279

**Published:** 2023-05-21

**Authors:** Amirhossein Aghakhani, Jaleh Shoshtarian Malak, Zahra Karimi, Fardis Vosoughi, Hojjat Zeraati, Mir Saeed Yekaninejad

**Affiliations:** ^1^ Department of Epidemiology and Biostatistics, School of Public Health Tehran University of Medical Sciences Tehran Iran; ^2^ Department of Digital Health, School of Medicine Tehran University of Medical Sciences Tehran Iran; ^3^ Department of Orthopedics and Trauma Surgery, Shariati Hospital and School of Medicine Tehran University of Medical Sciences Tehran Iran

**Keywords:** COVID‐19, gradient boosting, machine learning, prediction

## Abstract

**Background and Aims:**

To explore the use of different machine learning models in prediction of COVID‐19 mortality in hospitalized patients.

**Materials and Methods:**

A total of 44,112 patients from six academic hospitals who were admitted for COVID‐19 between March 2020 and August 2021 were included in this study. Variables were obtained from their electronic medical records. Random forest‐recursive feature elimination was used to select key features. Decision tree, random forest, LightGBM, and XGBoost model were developed. Sensitivity, specificity, accuracy, F‐1 score, and receiver operating characteristic (ROC)‐AUC were used to compare the prediction performance of different models.

**Results:**

Random forest‐recursive feature elimination selected following features to include in the prediction model: Age, sex, hypertension, malignancy, pneumonia, cardiac problem, cough, dyspnea, and respiratory system disease. XGBoost and LightGBM showed the best performance with an ROC‐AUC of 0.83 [0.822−0.842] and 0.83 [0.816−0.837] and sensitivity of 0.77.

**Conclusion:**

XGBoost, LightGBM, and random forest have a relatively high predictive performance in prediction of mortality in COVID‐19 patients and can be applied in hospital settings, however, future research are needed to externally confirm the validation of these models.

## 1 INTRODUCTION

Coronavirus infectious disease (COVID‐19) which emerged 3 years ago is caused by severe acute respiratory syndrome coronavirus 2 (SARS‐CoV‐2). The first case of the disease was reported to the World Health Organization (WHO) on December 31, 2019.^1^ WHO declared COVID‐19 a pandemic due to its rapid global spread on March 11, 2020.^2^ Fighting this pandemic has become the top priority for every nation in the world ever since. COVID‐19 still poses a serious challenge worldwide even after 3 years, despite advancements in treatment and prevention strategies such as vaccines. More than 750 million confirmed cases and 6.5 million deaths were registered until January 16, 2023, among which more than 2.5 million cases occurred during the last 7 days.^3^ Mortality has not diminished completely, despite its reduction after the administration of vaccines. In total, 24,863 patients died worldwide during January 9 to 16, 2023.^3^ Research suggest that vaccine effectiveness decreases from 80% to 30% after 6 months.^4^, ^5^ More people can be at risk of contracting COVID‐19, hospitalization, and mortality in the future with fewer people being boosted.

Complex statistical approaches have been widely applied in various research fields such as identifying biomarkers^6^, ^7^ for cardiovascular disease^8^ and diabetes with advances in computational systems during the past two decades.

Machine learning (ML) is considered as a branch of artificial intelligence, which aims to identify and learn patterns in complex data. ML models are broadly utilized in healthcare to develop prognostic and diagnostic models.^9^, ^10^

Demonstrating the appropriateness of ML algorithms plays a critical role in applied healthcare. A large number of ML models have been developed to guide healthcare professionals in recent years. Such prediction models may simultaneously assist healthcare workers in diagnosing patients and identifying high‐risk patients who may need extra care.

Previous studies have utilized different ML models to predict COVID‐19 mortality based on various features, including clinical symptoms and demographic information.^11^, ^12^, ^13^, ^14^ However, some clinical features require further laboratory testing which can be time‐consuming and hinder the ability to predict mortality rates upon admission. Additionally, previous studies on predicting COVID‐19 outcomes based on comorbidities have been limited by small sample sizes.

Patients' hospital and medical records are regarded as valuable sources for obtaining information about their medical history and comorbidities. This study aims to demonstrate the effectiveness of ML models in predicting COVID‐19 mortality based on demographic features and comorbidities, as well as comparing their performance.

To achieve this aim, a data set was collected from the electronic medical records related to six academic hospitals utilizing the recursive feature elimination (RFE) method to find relevant features which could contribute to the outcome of COVID‐19 patients. The data set was applied after feature selection to build prediction models based on ML algorithms to classify the patients' outcome into death and survival groups. Validation analysis was performed to evaluate the predictive power of each model. This study seeks to demonstrate the ability of several ML models to predict COVID‐19 mortality to assist healthcare professionals and compare their performances.

This study utilized a larger sample size than most relevant literature and assessed various ML algorithms, including novel models such as extreme gradient boosting (XGB) and light gradient boosting machine (LightGBM). Furthermore, classic ML algorithms such as decision tree (DT) and random forest (RF) were used to build the models.

## 2 MATERIALS AND METHODS

### 2.1 Data and setting

The COVID‐19 data set was gathered from medical records related to six academic hospitals. Patients were admitted with COVID‐19 from March 2020 to August 2021. The data for all of the patients were anonymized. The institutional review board approved the current study (IR.TUMS.SPH.REC.1401.045).

Inclusion criteria were based on the coding of the International Classification of Disease 10th Revision (ICD‐10). Patients with u07.1 and u07.2 codes were included. The aforementioned codes indicate the presence of COVID‐19 based on laboratory testing and clinical data without laboratory testing, respectively. Patients with missing outcome data and those who died less than 24 h after admission were excluded. The final data contained 44,112 patients, among which 5560 cases died.

### 2.2 Variables

The collected data set includes demographic features such as sex and age, symptoms such as cough, fever, chest pain, dyspnea, and their medical background including comorbidities such as hypertension, diabetes, obesity, acute renal failure (ARF), chronic kidney disease (CKD), cardiac problems, hepatic failure, malignancy, history of pneumonia, respiratory system disease, and disease of the nervous system (Table 1). All of the variables, except age are coded as categorical. The outcome was the death of a patient with COVID‐19 during hospitalization.

**Table 1:** Confusion matrix for binary classification.

	True class		
Predicted class		Dead	Survived
	Dead	True positive (TP)	False positive (FP)
	Survives	False negative (FN)	True negative (TN)

### 2.3 Data analysis

**Figure 1:**
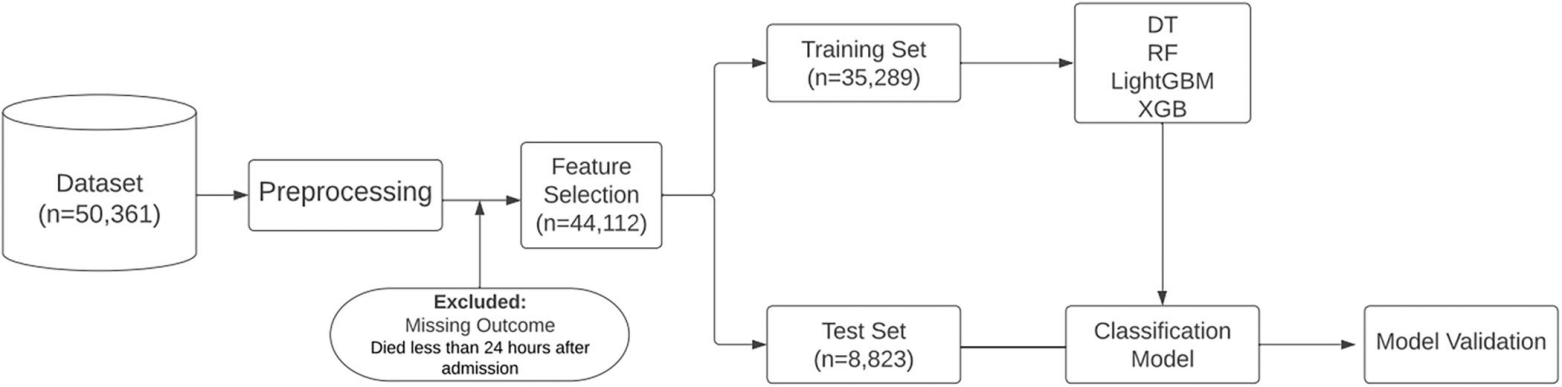
Workflow of the study. DT, decision tree; RF, random forest; XGB, extreme gradient boosting.

The Python programming language is used for preprocessing, feature selection, training, and evaluation of the models, as shown in Figure 1. In addition, the Scikit‐learn library^15^ is applied to split the data into a train and test set, as well as building and evaluating the presented model. Further, Pandas,^16^ NumPy,^17^ and Matplotlib^18^ libraries are used for data preprocessing and illustrating graphs.

First, the features observed in less than 0.2% of the patients were eliminated, resulting in the dropping of the ARF feature in the feature selection process due to sparsity.

Random forest RFE (RF‐RFE) method was utilized for feature selection.^19^ This iterative method trains a model, ranks features based on their significance, and eliminates the feature with lowest ranking. The above‐mentioned process resumes until a specific cut‐off or criterion such as selecting the minimum number of features is achieved.

The data set was split into train (80%) and test (20%) sets. Stratification was applied based on the death outcome to ensure the existence of enough samples from both outcomes in the aforementioned sets since the data is heavily imbalanced. In addition, class weight was used in data training to reduce the negative effect of data imbalances. The data in each class will have an inversely proportional weight of data frequency and gives more weight to each observation from the minority class. Further, the fivefold cross‐validation (CV) method was utilized for training the data and hyperparameter adjustment. The training data is split into fivefolds and trained in all except onefold which is used for validation. The final model with adjusted hyperparameters was applied to the test set to evaluate the effectiveness of the models. Randomized search CV was employed for hyperparameter tuning in XGBoost and LightGBM models. Furthermore, grid search CV was applied for RF and DT models.

Four ML models including DT, RF, XGB, and LightGBM were used to build a prediction model for mortality. Model performance was investigated by the area under the receiver operating characteristic (ROC) curve (AUC). Other evaluation metrics such as accuracy, sensitivity, specificity, and F‐1 score were acquired, as well.

DT is considered as a supervised nonlinear ML algorithm, which can be utilized for classification problems.

RF is regarded as an ensemble tree‐based method, which applies subsets of samples, as well as creating and aggregating multiple DTs to generate a predictive model.^20^, ^21^

Boosting is another ensemble method to improve DT predictions. Boosting uses and aggregates a series of weak learners to build a final prediction model.^20^, ^22^

Gradient boosting (GB) utilizes residual error for learning, which creates models to predict the error and adjusts the outcome prediction of the model based on error prediction. Learning rate in GB is regarded as constant throughout the whole process of model building. XGB uses GB to build a model applying a more regularized term to avoid overfitting and obtain a better performance.^23^

Generalizability of the model should be reviewed to study its performance after it is trained using test set data. The proposed model predicts the outcome of patients in the test set. Then, the predicted outcome related to each patient with its true outcome is compared. To utilize the evaluation metrics, four counts including true positives (TP), false positives, true negatives (TN), and false negatives are applied (Table 1). Now, the basic performance metrics including sensitivity, specificity, accuracy, and F‐1 score can be achieved with the following formulas. Sensitivity and specificity evaluate how well models can predict TP and TN of each outcome, respectively. Accuracy is another metric that shows what percentage of data was predicted correctly by models. F‐1 score is another metric that evaluates the accuracy of the model that incorporates both recall (sensitivity and specificity) and precision (positive and negative predictive value).

(1) $$\mathrm{Sensitivity}=\frac{\mathrm{TP}}{\mathrm{TP}+\mathrm{FN}}$$

(2) $$\mathrm{Specificity}=\frac{\mathrm{TN}}{\mathrm{FP}+\mathrm{TN}}$$

(3) $$\mathrm{Accuracy}=\frac{\mathrm{TP}+\mathrm{TN}}{\mathrm{TP}+\mathrm{FN}+\mathrm{FP}+\mathrm{TN}}$$

(4) $$F-1\mathrm{score}=\frac{2\mathrm{TP}}{2\mathrm{TP}+\mathrm{FP}+\mathrm{FN}}$$

## 3 RESULTS

The data of 50,361 COVID‐19 patients were collected, among which 44,112 patients met the inclusion criteria and 6249 patients were excluded from the data. The final cohort contained patients that admitted to hospitals during March 2020 to August 2021. In total, 5560 (12.6%) patients out of the 44,112 died. The cohort included 20,065 (45.5%) females. The mean age was 53.9 ± 17.5 years. The descriptive statistics for the 44,112 samples in the data set are presented in Table 2.

**Table 2:** Sample characteristics.

	Survived (*n* = 38,552)	Death (*n* = 5560)	Overall (*n* = 44,112)
Age			
Mean (SD)	52.1 (17.0)	66.9 (15.6)	53.9 (17.5)
Sex			
Female	17,803 (46.2%)	2262 (40.7%)	20,065 (45.5%)
Male	20,749 (53.8%)	3298 (59.3%)	24,047 (54.5%)
Hypertension			
No	36,586 (94.9%)	4711 (84.7%)	41,297 (93.6%)
Yes	1966 (5.1%)	849 (15.3%)	2815 (6.4%)
Diabetes			
No	36,264 (94.1%)	4671 (84.0%)	40,935 (92.8%)
Yes	2288 (5.9%)	889 (16.0%)	3177 (7.2%)
Obesity			
No	38,490 (99.8%)	5521 (99.3%)	44,011 (99.8%)
Yes	62 (0.2%)	39 (0.7%)	101 (0.2%)
Acute renal failure			
No	38,531 (99.9%)	5509 (99.1%)	44,040 (99.8%)
Yes	21 (0.1%)	51 (0.9%)	72 (0.2%)
Chronic kidney disease (CKD)			
No	37,413 (97.0%)	5064 (91.1%)	42,477 (96.3%)
Yes	1139 (3.0%)	496 (8.9%)	1635 (3.7%)
Malignancy			
No	37,948 (98.4%)	5127 (92.2%)	43,075 (97.6%)
Yes	604 (1.6%)	433 (7.8%)	1037 (2.4%)
Cardiac problem			
No	37,528 (97.3%)	4934 (88.7%)	42,462 (96.3%)
Yes	1024 (2.7%)	626 (11.3%)	1650 (3.7%)
History of pneumonia			
No	34,358 (89.1%)	3672 (66.0%)	38,030 (86.2%)
Yes	4194 (10.9%)	1888 (34.0%)	6082 (13.8%)
Hepatic failure			
No	38,448 (99.7%)	5479 (98.5%)	43,927 (99.6%)
Yes	104 (0.3%)	81 (1.5%)	185 (0.4%)
Fever			
No	35,575 (92.3%)	5186 (93.3%)	40,761 (92.4%)
Yes	2977 (7.7%)	374 (6.7%)	3351 (7.6%)
Cough			
No	33,658 (87.3%)	4860 (87.4%)	38,518 (87.3%)
Yes	4894 (12.7%)	700 (12.6%)	5594 (12.7%)
Dyspnea			
No	32,277 (83.7%)	4382 (78.8%)	36,659 (83.1%)
Yes	6275 (16.3%)	1178 (21.2%)	7453 (16.9%)
Chest pain			
No	37,442 (97.1%)	5433 (97.7%)	42,875 (97.2%)
Yes	1110 (2.9%)	127 (2.3%)	1237 (2.8%)
Disorder of lipoprotein metabolism			
No	38,379 (99.6%)	5517 (99.2%)	43,896 (99.5%)
Yes	173 (0.4%)	43 (0.8%)	216 (0.5%)
Nervous system disease			
No	38,153 (99.0%)	5376 (96.7%)	43,529 (98.7%)
Yes	399 (1.0%)	184 (3.3%)	583 (1.3%)
Respiratory system disease			
No	37,582 (97.5%)	4331 (77.9%)	41,913 (95.0%)
Yes	970 (2.5%)	1229 (22.1%)	2199 (5.0%)

History of pneumonia is considered as the most prevalent feature among patients who died (*N* = 1888). In addition, 4194 (10.9%) patients in survived group exhibit a history of pneumonia.

**Figure 2:**
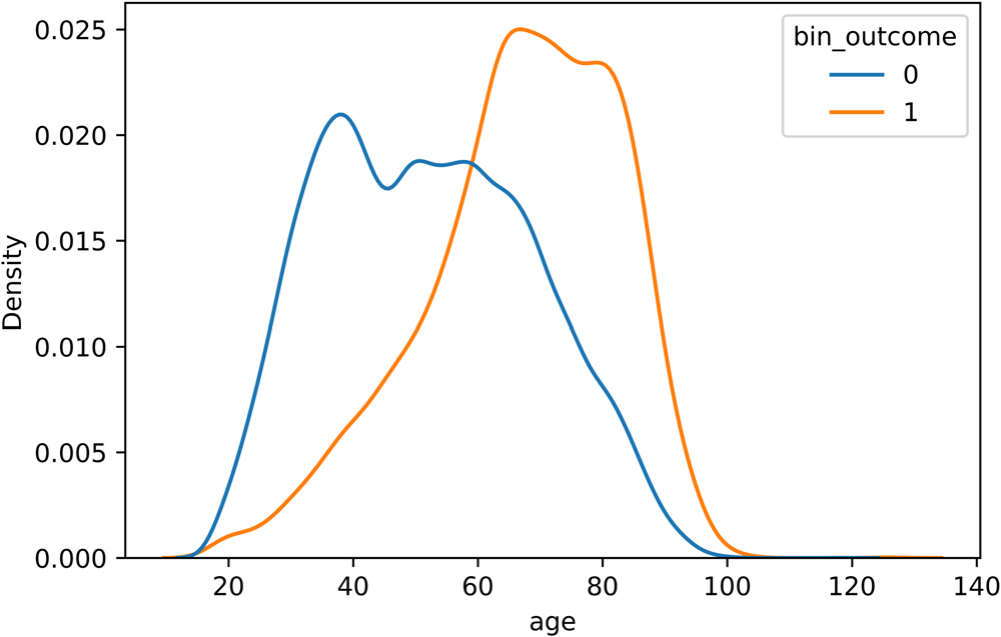
Density plot of age for different outcomes (bin_outcome = 0 indicates the survived group).

A significant difference is reported between groups in terms of age. Median and mean (SD) of age equal 51 and 52.1 (17.0%) for survived group, and 69 and 66.9 (15.6%) for the patients who died, respectively. The density plot (Figure 2) of age also capitalizes this difference.

As shown in Table 2, the death group exhibits a higher proportion of patients with hypertension. In addition, 2288 (5.9%) patients in survived group and 849 (15.3%) ones in death group suffer from hypertension, repectively.

Prevalence of CKD in the death group is three times greater than the survived one. Based on the results, 1139 (3.0%) patients in survived group and 496 (8.9%) in the death group suffer from CKD, respectively.

Overall, the prevalance proportion of comorbidities is higher in the death group. However, most symptoms such as cough, fever, and chest pain are more proportionally prevalant in the survived group.

**Figure 3:**
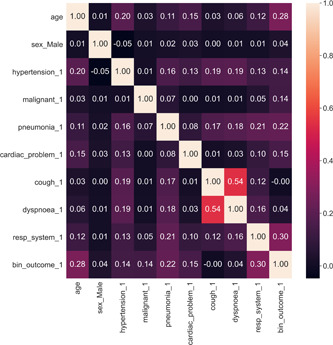
Correlation matrix of included features.

Figure 3 illustrates the correlation coefficient between variables. The highest correlation between features is observed between cough and dyspnea (*r* = 0.54). Additionally, respiratory system (*r* = 0.3) and cough (*r* = 0.28) exhibit the highest correlation with the outcome variable.

RF‐RFE was applied for feature selection and minimum features to select was set at nine features. Following features were selected to include in the model: Age, sex, hypertension, malignancy, pneumonia, cardiac problem, cough, dyspnea, and respiratory system disease.

CV was performed to acquire the best hyperparameters in each model. Gini impurity was used in the DT as the split criterion. Max depth and the minimum sample for leaf and split equals five. As represented in Table 3, the DT displays an AUC, sensitivity, and specificity of 0.68, 0.69, and 0.67, respectively.

**Table 3:** Performance of DT, RF, LightGBM, and XGBoost methods in predicting COVID‐19 mortality.

Model	ROC‐AUC	95% CI for AUC	Sensitivity	Specificity	Accuracy	F1 score
Decision tree	0.68	0.664−0.694	0.69	0.67	0.67	0.73
Random forest	0.81	0.803−0.824	0.71	0.77	0.77	0.80
LightGBM	0.83	0.816−0.837	0.74	0.77	0.77	0.80
XGBOOST	0.83	0.822−0.842	0.74	0.77	0.76	0.80

Abbreviations: DT, decision tree; RF, random forest; ROC, receiver operating characteristic.

The maximum features to create a tree are set as the square root of the number of features in RF. Minimum sample for leaf and split equals five. Max depth and number of estimators are set at 20. RF shows an AUC, sensitivity, and specifity of 0.81, 0.71, and 0.77, respectively.

**Figure 4:**
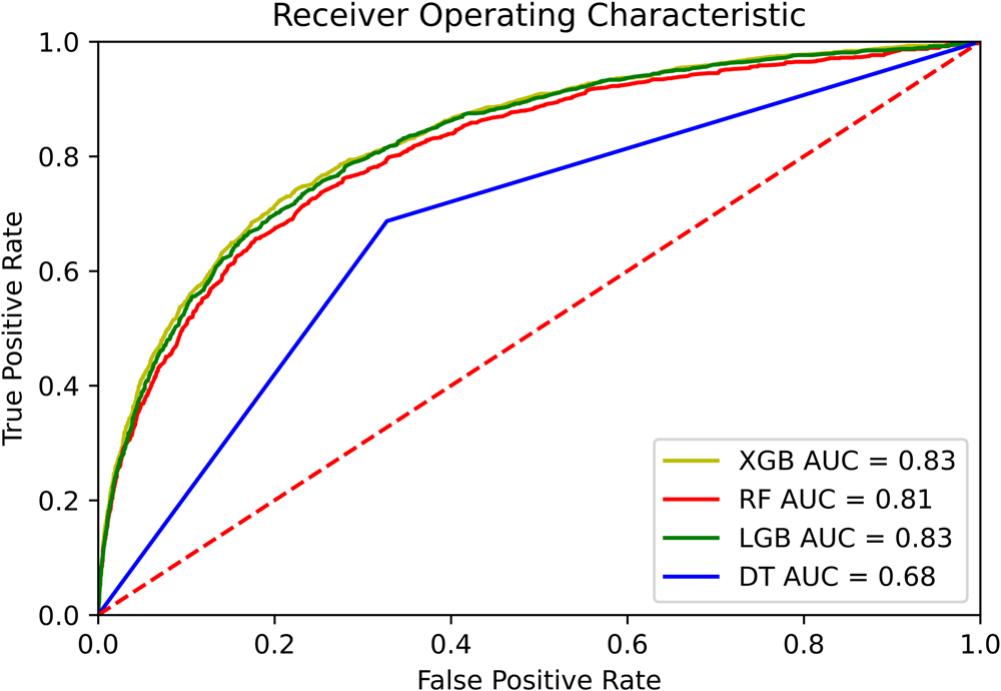
Comparison of ROC curves between models. ROC, receiver operating characteristic.

Learning rate and max depth are set at 0.1 and 3 in XGB, respectively. These values for LightGBM and max depth equals 29. Both XGB and LightGBM yield similar results with an AUC of 0.83. Figure 4 compares different models in prediction with the ROC curve. Table 4 presents the results related to tuned hyperparameters utilized to train the models.

**Table 4:** Hyperparameters selected to train the model after CV.

Model	Hyperparameters
Decision tree	“criterion”= “gini”,“max_depth”= 5,“max_features”= 5,“min_impurity_decrease”= 0.05,“min_samples_leaf”= 20,“min_samples_split”= 5
Random forest	“max_depth”= 20,“max_features”= “sqrt”,“min_samples_leaf”= 5,“min_samples_split”= 5,“n_estimators”= 60
LightGBM	“num_leaves”= 144,“n_estimators”= 650, “min_split_gain”= 0.05,“max_depth”= 29,“learning_rate”= 0.1,“colsample_bytree”= 0.55
XGBoost	“min_child_weight”= 1,“max_depth”= 3,“learning_rate”= 0.1,“gamma”= 0.3,“colsample_bytree”= 0.5

Abbreviation: CV, cross‐validation.

## 4 DISCUSSION

This study aimed to build several different prediction models by applying different ML algorithms to compare such models for predicting COVID‐19 mortality. These records and features were used because medical records related to each patient can be collected easily. Even if those records are not available, the selected features can easily be obtained and can simply be asked from patients. In addition, by using medical record data, the outcome of patients can be predicted upon admission to the hospital due to the lack of need for laboratory testing.

**Figure 5:**
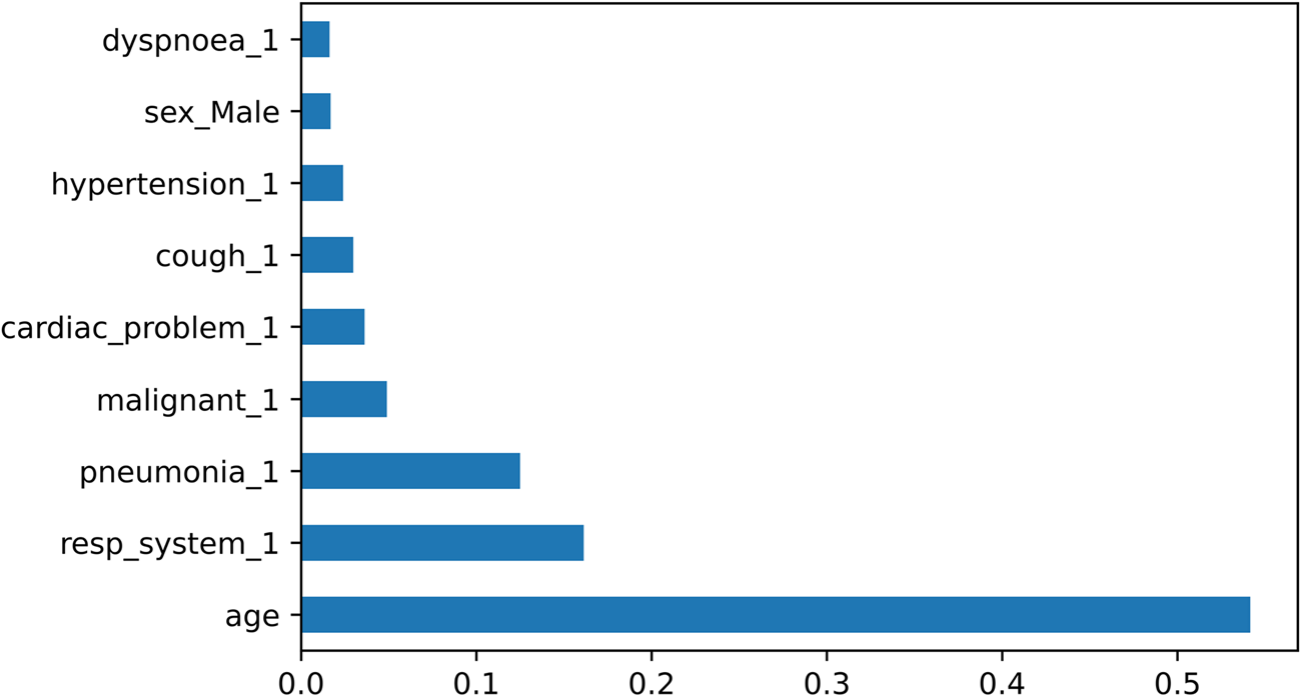
Feature importance based on the random forest model.

XGBoost, LightGBM, and RF demonstrated the best predictive performances among all of the built prediction models. RF displayed an AUC, sensitivity, and specificity of 0.81, 0.71, and 0.77, respectively. As shown in Figure 5, age, respiratory system disease, and history of pneumonia and malignancy are among the most significant features in the RF model. XGB and LightGBM exhibit similar performance with AUC and sensitivity of 0.83 and 0.74, respectively. In addition, DT demonstrates the weakest performance with an AUC of 0.68. All of the results were obtained from the test set.

RF shows a notably better performance than the DT due to several reasons. DTs are regarded as non‐robust, meaning that even a minor alteration in data can create a huge difference in the final model. Bagging, RFs, and boosting are proposed to eliminate these obstacle.

Bagging utilizes subsets of samples to build multiple trees and takes a majority vote between trees to classify each observation. Bagging uses all of the available features to build each tree which results in increased correlation between the trees and and thus it does not contribute a major role to reducing the variance.

RF improves bagging using a subset of features for splitting and building a tree, leading to a de‐correlation and more difference between trees and hence averaging is more reliable and reduces the variance more than the two previous methods. Boosting is another method to improve DT results.

Boosting is similar to bagging. However, the trees are considered independent in bagging whereas in boosting trees are built in a sequence, meaning that each tree learns and corrects the previously built tree.^20^, ^24^

The aforementioned models except DT exhibit relatively high AUC‐ROC of 0.81 and 0.83 utilizing only demographic features, medical history, and comorbidities of the patients. These ML models demonstrated that patients who are more likely to die can be appropriately identified upon their admission to hospitals. Based on these models, more than 70% of patients that die can be identified with only limited sets of variables and without conducting a single laboratory test. Therefore, such models are considered as practical for predicting mortality among COVID‐19 patients. Predicting the patients' outcome upon their admission can be useful in hospital‐settings, especially in low and middle‐income countries where staff, drug, and bed shortages often occur. Therefore, high‐risk patients can be identified and prioritized by applying these ML models.

Several models were previously offered to predict mortality using laboratory results. However, acquiring such models needs further testing and can be time‐consuming. Thus, mortality cannot be predicted upon admission using the aforementioned features. Kim et al.^13^ utilized demographic and clinical information related to 13,190 patients to develop a model to rapidly predict COVID‐19 patients' mortality. They proposed further testing of models in different populations and cohorts. Additionally, most of their data were from the pandemic's first wave.^13^ In comparison, this study only uses case‐history data and is able to predict mortality status more rapidly. Although, this comes at a cost of a slight decrease in prediction power. The data set of this study also benefited from patients from a wider range of time which included the first wave of the pandemic and those from which other variants such as delta were regarded as the dominant one. Such inclusion of different variants helps the generalizability of the models.

In addition, Estiri et al. built only a GB model using electronic medical records as features.^25^ In comparison, this study used a bigger sample size and a smaller feature set that can easily be obtained. Further, Klen et al.^26^ designed a COVID‐19 disease outcome predictor (CODOP) by applying a mixture of laboratory and clinical information, as well as performing the model on a multinational sample. However, their sample only included patients from America and Europe and did not include patients from Asia. Laboratory data of these studies can obey different protocols country by country or even center by center, creating a limitation to include such features in their prediction model, while medical records and comorbidities follow a universal protocol and are coded as International Classification of Disease (ICD‐10).^27^ Therefore, such models can easily be applied and tested in other populations without the above‐mentioned limitations.

This study has a number of limitations. First, this study uses hospital‐based data and only includes patients admitted to the hospital, and did not include other COVID‐19 patients. Mortality can differ from population to population based on genetics and lifestyle. Further studies in different populations should be conducted to eliminate the aforementioned effect and validate the generalizability of the models. In addition, this study does not consider all of the covariates such as progress in the treatment of COVID‐19, differences in patients with different variants, and discrepancies in hospital services, which may affect mortality. The data was collected when vaccines were in the first step of administration in Iran and only 16% received their first dose. Therefore, vaccination status could not be included in the model. Vaccination is hypothesized to be a helpful index although previous studies indicated that such process reduces hospital admission up to 94%.^28^ Finally, although laboratory results were not included in this study, these results are a useful tool to evaluate patients' prognoses after they are admitted to the hospital and can be incorporated into future models.

## 5 CONCLUSIONS

This study indicated that COVID‐19 mortality can be predicted from a limited set of features that can easily be obtained utilizing ML algorithms. XGBoost and LightGBM yielded the best results with a ROC‐AUC of 0.83 [0.822−0.842] and 0.83 [0.816−0.837], respectively. The present study did not require laboratory and complex test results to predict the mortality rate. However, future studies should validate such models by applying different data sets from various cohorts.

## AUTHOR CONTRIBUTIONS

**Amirhossein Aghakhani**: Conceptualization; data curation; formal analysis; investigation; methodology; software; visualization; writing—original draft. **Jaleh Shoshtarian Malak**: Conceptualization; formal analysis; investigation; resources; software; validation. **Zahra Karimi**: Conceptualization; data curation; investigation; software; validation; visualization. **Fardis Vosoughi**: Methodology; supervision; writing—original draft. **Hojjat Zeraati**: Conceptualization; formal analysis; methodology; project administration; supervision; writing—original draft. **Mir Saeed Yekaninejad**: Conceptualization; project administration; resources; software; supervision; validation; visualization; writing—review and editing.

## CONFLICT OF INTEREST STATEMENT

The authors declare no conflict of interest.

## ETHICS STATEMENT

This study was approved by the ethics committee of Tehran University of Medical Sciences (IR.TUMS.SPH.REC.1401.045). This study was approved by institutional review board of Tehran University of Medical Sciences (IR.TUMS.SPH.REC.1401.045).

## TRANSPARENCY DECLARATION

The lead author (Hojjat Zeraati and Mir Saeed Yekaninejad) affirms that this manuscript is an honest, accurate, and transparent account of the study being reported; that no important aspects of the study have been omitted; and that any discrepancies from the study as planned (and, if relevant, registered) have been explained.

## Data Availability

The data sets analyzed during the current study are available from the corresponding author upon reasonable request.
